# Investigated Different Ratios of Cotton Seed Cake With Dried *Sesbania sesban* Leaves on the Feed Intake, Digestibility and Growth Performance of Semien Sheep

**DOI:** 10.1002/vms3.70862

**Published:** 2026-03-02

**Authors:** Wondimu Demoz Tessema, Aschalew Assefa Teshager, Shewangzaw Addisu Mekuria

**Affiliations:** ^1^ College of Veterinary Medicine and Animal Sciences University of Gondar Gondar Ethiopia

**Keywords:** cotton seed cake, natural pasture hay, Semien sheep, *Sesbania sesban*, supplement

## Abstract

**Background:**

Currently the cost and availability of protein‐source feed for fattening of sheep is challenging.

**Objectives:**

This study investigated different ratios of cotton seed cake with dried *Sesbania sesban* leaves on the feed intake, digestibility and growth performance of Semien sheep.

**Methods:**

The experiment was conducted for 90 days with 7 days of digestibility trials. A total of 20 intact male Semien lambs with an initial body weight of 15.16 ± 0.96 were used. A randomized complete block design was used with five treatments: T1 = 100% cotton seed cake (CSC), T2 = 75% CSC + 25% SSL, T3 = 50% CSC + 50% SSL, T4 = 25% CSC + 75% SSL and T5 = 100% SSL were offered 300 g/day as feed basis, and the natural pasture hay and water were given ad libitum. Data on feed intake, body weight change and digestibility were subjected to analysis of variance (ANOVA) using statistical analysis system (SAS) 20.0.

**Results:**

The lowest (*p* < 0.01) hay dry matter (DM) intake was recorded for T1. The total DM intake was highly significant (*p* < 0.01) among treatment groups in the order of T4 > T3 = T5 > T2 > T1. The apparent digestibility of DM, organic matter (OM) and crude protein (CP) was highly significant (*p* < 0.001) among treatment groups. There were high records (*p* ≤ 0.001) of body weight change at T3 and low in T1. The partial budget analysis showed that the net return of T3, T2, T1, T4 and T5 was 1024, 990.37, 903 and 790.12 ETB/treatment, respectively.

**Conclusion:**

The findings showed that from biological and economic responses, sheep fed on 50% CSC + 50% SSL could be recommended.

## Graphical abstract

The price of feed cost is one of the main challenges for animal producers in Ethiopia. Sesbania is not utilized for animal feed and is commonly found in different geographical locations. Therefore, mixing of cotton seed cake with dried *Sesbania sesban* leaves at equal proportion improves biological performance of Semien sheep and economically visible.

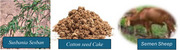

## 1 Introduction

Ethiopia has a large livestock population, with an expected number of 70 million cattle, 52.5 million goats, 42.9 million sheep and 57 million chickens (CSA 2021). Sheep play a crucial role in livestock farming, being essential to household livelihood and making a significant contribution to Ethiopia's national economy (Mekuriaw and Asmare 2018). Moreover, the demand for animal protein has increased due to the high global human population increase (Mekuria et al. 2024).

Sheep production in Ethiopia plays a very important role in contributing to food security, domestic meat consumption, generating cash income and providing continuous service to the economic stability of smallholder farmers (Mathewos et al. 2021). Because of their high fertility, short generation interval, ability to thrive in harsh environments and capacity to produce with limited feed resources, sheep production is thought to be advantageous when compared to other classes of livestock production (Anteneh and Yadav, 2017).

Nutritionally speaking, lamb meat is a rich source of minerals, including iron, zinc, calcium, selenium, potassium, copper and magnesium, as well as various B vitamins (Boada et al. 2016). Consuming lamb meat offers significant advantages such as protecting against anaemia, enhancing muscle mass, promoting optimal bone health, boosting the immune system and nervous system, as well as preventing birth defects, among other benefits (Pogorzelska‐Nowicka et al. 2018). Most sheep in Ethiopia are slaughtered at about 8–12 months of age and have a body weight of only 28 kg (Gashu et al. 2017). This shows that there is scope for improvement in sheep performance by improving feeding, productive management practices and health care management (Ayele and Urge 2018).

Feed resources in Ethiopia, stump grazing and natural pasture serve as the primary sources of feed for sheep (Aleme 2022). In a mixed‐crop livestock production system, one strategy to increase the feeding value of animals is feed supplementation. This involves utilization of fodder banks, fodder trees and agro‐industrial products such as noug seed cake and urea to overcome crude protein (CP) shortages (Yirdaw et al. 2017a). Agro‐industrial by‐products are inaccessible and unaffordable to smallholder farmers; hence, alternative supplementary feedstuffs produced on farms other than commercial concentrates are essential (Yirdaw et al. 2017b). To address the issue of protein shortage in low‐quality feeds during times of feed scarcity, it may be possible to strategically supplement underutilized, inexpensive, less competitive, year‐round available and easily accessible protein sources like *Sesbania sesban* (Tekliye et al. 2018). With a high non‐degradable protein value, cotton seed cake (CSC) is a high‐protein feed for cattle (Barman et al. 2019). CSC is an excellent protein supplement for fattening lambs and is practically equal to linseed meal for fattening lambs (Michaele 2018). Sheep's intake of total dry matter (DM), organic matter (OM) and CP increased when *S. sesban* leaves were added (A. Mekoya et al. 2009). Additionally, the average daily body weight gain, carcass characteristics and feed conversion efficiency all showed significant improvements when *S. sesban* was added as a supplement (Tekliye et al. 2018). Therefore, the objective of this study to investigate different ratios of CSC with dried *S. sesban* leaves on the feed intake, digestibility and growth performance of Semien sheep.

## 2 Materials and Methods

### 2.1 Experimental Site

**FIGURE 1:**
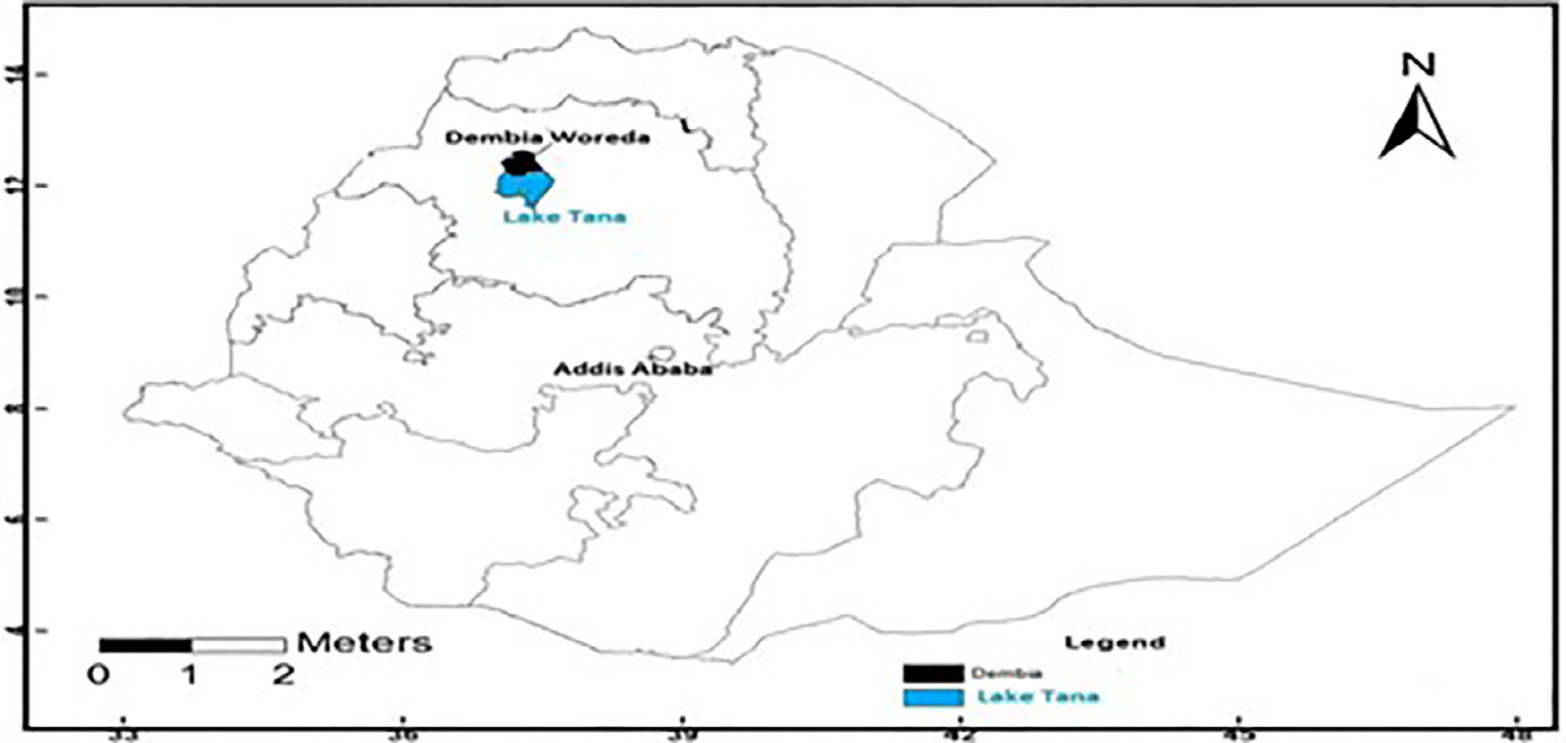
Map of the study area source (Teshome 2016).

The study was conducted in East Dembia district of the central Gondar zone, Ethiopia. It is located 750 km northwest of Addis Ababa and 35 km southwest of Gondar at a latitude and longitude of 12°17′ N and 37°26′ E, respectively (BFED, 2018) and at an elevation ranging from 1500 to 2600 m.a.s.l. with an annual minimum and maximum temperature of 17°C and 28°C, respectively. The average annual rainfall of the area was 700–1200 mm reported by East Dembia Woreda Office of Agriculture (2012) (Teshome 2016). Figure 1 shows a map of the study area.

### 2.2 Experimental Animal Management

A total of 20 intact male sheep with an initial body weight of 15.16 ± 0.96 (mean ± SD) were purchased from the local market in Wogera, Ethiopia. The age of the sheep was determined by taking information from the sheep owner and observing dentition. All of the sheep had ear tags applied before the experiment started in order to identify them and de‐wormed ivermectin and albendazole for external and internal parasites, respectively. The animals were acclimatized for 15 days followed by 90 days of consecutive feeding trial and 7 days of digestibility trials. The animals were kept in well‐ventilated individual pens (1 m by 1.5 m) equipped with a separate watering and feeding trough. Throughout the trial, all of the animals were closely monitored to see if any illnesses or disorders developed.

### 2.3 Experimental Feed Preparation and Feeding

Feed ingredients used for experimental ration preparation were a different ratio of cotton seed cake and *S. sesban* leaf meal. The *S. sesban* was collected from Dembia Woreda, and the leaves were cute at a tree height of around 2 m. Then, the leaves were air‐dried under a shed, packed in sacks and stored until feeding. The supplement concentrates (CSC and *S. sesban*) were weighed separately and thoroughly mixed at a different ratio before being offered. Grass hay was chopped into smaller sizes (≈5 cm) and fed ad libitum (allowing ≈20% refusal). The daily supplements were fed into two equal portions at 08:00 and 16:00 h in a separate container. Each experimental animal was given 300 g of feed basis daily with different proportions.

### 2.4 Experimental Design and Treatments

Twenty sheep were randomly assigned to five feed treatments, based on their initial live body weight. A randomized complete block design (RCBD) was used with the five treatments and four replications. The mixed ration was considered isonitrogenous, and the total daily offer of *S. sesban* with CSC mixture was 300 g/day/individual as shown in Table 1.

**TABLE 1:** Experimental feeds and natural pasture hay.

Treatments	Experimental feeds (%)	
	CSC	*S. sesban*
T1	100	0
T2	25	75
T3	50	50
T4	75	25
T5	0	100

Abbreviations: CSC = cotton seed cake; *S. sesban* = *Sesbania sesban;* T = treatment.

### 2.5 Data Collection

#### 2.5.1 Feed Intake

The amount of feed offered and refused for each sheep was recorded daily throughout the experimental period. Daily feed intake of experimental animals was calculated on a feed basis as the difference between the feed offered and refused.

#### 2.5.2 Body Weight Measurement

The initial and final body weights of animals were taken twice on two consecutive days, and the average of the two was taken as initial and final weights, after overnight fasting, respectively, with a Salter balance. The live weight change was calculated as the difference between final and initial body weights. Average daily weight gain was calculated as a weight gain divided by the number of feeding days:

$$\mathrm{BWC}=\mathrm{Final}\;\mathrm{Body}\;\mathrm{Weight}\left(\mathrm{FBW}\right)-\mathrm{Initial}\;\mathrm{Body}\;\mathrm{Weight}\left(\mathrm{IBW}\right)$$

$$\begin{array}{ccc} & & Average\;daily\;weight\;gain\;(ADGkg/d)\\ & & =\frac{\mathrm{Body}\;\mathrm{weight}\;\mathrm{gain}}{\mathrm{Number}\;\mathrm{of}\;\mathrm{feeding}\;\mathrm{days}} \end{array}$$

#### 2.5.3 Feed Conversion Efficiency

Feed conversion efficiency is used to know how efficiently the sheep convert the feed into body weight. It was measured using the formula suggested by Ferreira et al. (2021):

$$\begin{array}{ccc} & & \mathrm{Feed}\;\mathrm{conversio}\;\mathrm{efficiency}\left(\mathrm{FCE}\right)\\ & & =\frac{\mathrm{Average}\;\mathrm{daily}\;\mathrm{live}\;\mathrm{weight}(g)}{\mathrm{Average}\;\mathrm{daily}\;\mathrm{feed}\;\mathrm{intake}(g)}. \end{array}$$

### 2.6 Digestibility Trial

After 90 days of the feeding experiment, a digestibility trial was conducted. The 3 days of adaptation for carrying a faecal bag were followed by a faecal collection for seven consecutive days. Faeces were collected, weighed and recorded every morning from each animal before giving feed or water. A total of 20% of sample faeces were collected from the daily collection, kept in airtight plastic containers and frozen at −20°C until the chemical analysis was done. At the end of the experiment, the faecal sample of each sheep was taken and partially dried at 65°C for 72 h, and then the samples were grounded and stored in an airtight plastic container. The apparent digestibility coefficient (DC) of DM, OM, CP, crude fibre (CF), acid detergent fibre (ADF) and acid detergent lignin (ADL) was calculated using the following equation (McDonald et al. 2002):

$$\begin{array}{ccc} & & \;Apparentnutrient\;digestibility(\%)\\ & & =\frac{\mathrm{Nutrient}\;\mathrm{intake}\;-\;\mathrm{Nutrient}\;\mathrm{in}\;\mathrm{faces}}{\mathrm{Nutrient}\;\mathrm{intake}}\times 100 \end{array}$$

### 2.7 Chemical Analysis

The samples collected from daily feed offered and refused, including the faeces from each treatment, were analysed for DM, OM, ash, CP, NDF, ADF and ADL. The DM, OM, ash and nitrogen (N) were analysed according to the procedures of AOAC (2005). CP was measured by the Kjeldahl method as N × 6.25. NDF, ADF and ADL were analysed by the method (Van Soest 2020).

### 2.8 Partial Budget Analysis

A partial budget analysis was performed using the procedure of Upton (1979). Variable costs (feed cost) and selling and purchasing price of experimental sheep were included in the calculation, but other costs, such as labour for feed collection, pen cleaning and feeding experimental animals, were similar and not included in the calculations. The total return (TR) was calculated as the difference between the selling and purchasing prices of the experimental animals.

Net return (NR) was calculated as the amount of money left when total feed cost (TVC) was subtracted from TR:

NR=TR−TVC

The change in NR (ΔNR) was calculated as the difference between the change in TR (ΔTR) and the change in total variable costs (ΔTVC):

ΔNR=ΔTR−ΔTVC

The marginal rate of return (MRR) measures the increase in NR (ΔNR) associated with each additional unit of expenditure (ΔTVC) and is expressed in percentage as

$$\mathrm{MRR}=\Delta \mathrm{NR}/\Delta \mathrm{TVC}\times \left(100\right)$$

### 2.9 Statistical Analysis

Data on feed intake, body weight change and digestibility were subjected to an analysis of variance (ANOVA) model for RCBD using statistical analysis system (SAS) version 9.4 software. The differences in the treatment means were considered significant at the probability level of *p* < 0.05. Significantly different treatment means were compared by using LSD (least significant difference test).

The model used for this study was

Yij=μ+Ti+Bj+εij

where 𝑌𝑖𝑗 is the response variable, *μ* is the overall mean, 𝑇𝑖 is *i*th treatment, 𝛽𝑗 is the *j*th block effect, 𝜀𝑖𝑗 is the random error.

## 3 Results

### 3.1 Chemical Analysis of the Experimental Feeds

The chemical composition of the experimental feed is shown in Table 2. The CP content of treatment diets CSC, *S. sesban* and their mixture was higher than that of hay, whereas the fibre fraction of NDF, ADF and ADL in the treatment mixture was relatively lower than that of basal feed. CSC and *S. sesban* have approximately parallel values of CP, NDF and ADF.

**TABLE 2:** Chemical composition of experimental feed.

Chemical composition % DM							
Feed offered	DM	OM	CP	NDF	ADF	ADL	Ash
Hay	93.00	83.32	6.00	53.79	36.56	6.50	9.68
T1	94.00	89.74	27.28	49.78	34.57	5.96	4.26
T2	93.25	87.31	26.75	49.88	34.72	6.05	5.94
T3	92.5	84.8	26.19	49.98	34.87	6.14	7.63
T4	91.75	82.44	25.67	50.08	35.01	6.23	9.31
T5	91.00	80.01	25.13	50.18	35.16	6.32	10.99
Hay refusal							
T1	94.00	84.43	5.00	71.57	59.67	10.92	9.57
T2	93.00	80.10	5.34	64.47	53.76	8.99	12.90
T3	94.00	84.43	5.20	65.99	53.19	9.22	9.57
T4	93.00	84.40	4.66	73.12	61.70	11.08	8.60
T5	94.00	86.55	5.37	58.90	47.13	7.99	7.45

*Note*: T1 = only 100% CSC; T2 = 75% CSC + 25% *S. sesban*; T3 = 50% CSC + 50% *S. sesban*; T4 = 25% CSC + 75% *S. sesban*; and T5 = only 100% *S. sesban*.

Abbreviations: ADF = acid detergent fibre; ADL = acid detergent lignin; CP = crude protein; CSC = cotton seed cake; DM = dry matter; NDF = neutral detergent fibre; OM = organic matter; *S. sesban* = *Sesbania sesban*.

### 3.2 DM and Nutrient Intake

The daily DM and nutrient intake of Semien sheep are indicated in Table 3. There was no refusal of intake of concentrate feed to the experimental animals. There was a statistically significant difference (*p* < 0.01) in hay DM intake between treatments. Hay DM intakes (g/day) of experimental sheep fed on T4 were the highest followed by T3 = T5, T2 and T1. The lower hay DM intake for T1 could be associated with the fact that CSC has relatively low rumen degradability due to its high fibre content. The total DM, NDF and ADF, in the present study, were highly significant differences (*p* < 0.01) among all treatment groups. Moreover, OM had significant differences (*p* < 0.05) between all treatment groups. However, CP intake was not significant difference (*p* > 0.05) between all treatment groups.

**TABLE 3:** Daily dry matter and nutrient intakes of Semien sheep fed hay and supplemented with a concentrate mixture, cotton seed cake (CSC) and *Sesbania sesban* leaves.

Parameters	Treatment feeds						
	T1	T2	T3	T4	T5	SEM	SL
DM (g/day)							
Hay DMI	444.72^c^	506.64^bc^	561.88^ab^	587.54^a^	549.72^ab^	14.26	⁎⁎
Supplement DM	300	300	300	300	300	_—_	_—_
Total DMI	744.72^c^	806.64^bc^	861.88^ab^	887.54^a^	849.72^ab^	14.26	⁎⁎
Total nutrient intake (g/day)							
OMI	667.65^b^	715.83^ab^	758^a^	773.75^a^	732.5^a^	11.36	⁎
CPI	110.52	112.90	114.86	114.90	110.85	1.12	ns
NDFI	406.55^c^	442.66^ab^	474.92^ab^	490.03^a^	468.49^ab^	8.31	⁎⁎
ADFI	278.35^c^	303.32^bc^	325.44^ab^	336^a^	321.58^ab^	5.70	⁎⁎

*Note*: Mean values in a row having different superscripts (a–c) differ significantly; ** = significant at *p* < 0.01; * = significant at *p* < 0.05, T1 = only 100% CSC; T2 = 75% CSC + 25% *S. sesban*; T3 = 50% CSC + 50% *S. sesban*; T4 = 25% CSC + 75% *S. sesban*; and T5 = only 100% *S. sesban*.

Abbreviations: ADFI = acid detergent fibre intake; CPI = crude protein intake; DMI = dry matter intake; NDFI = neutral detergent fibre intake; ns = not significant; SEM = standard error of the mean; SL = significance level.

In the present study, the total DM and OM intakes appeared to be highly impacted by the proportion of the supplemental DM intake. The total DM intakes were lowest for T1, intermediate for T2 and highest for the other three treatments, T3, T4 and T5 (*p* < 0.01). Similarly, the total OM intakes were lowest for T1 and T2; intermediate for T5 and highest for T3 and T4 at *p* < 0.05.

### 3.3 Apparent DM and Nutrient Digestibility

The mean apparent digestibility of DM and nutrients in Semien sheep is shown in Table 4. There was highly significant difference (*p* < 0.001) in DM, OM and CP digestibility at *p* < 0.001, among treatments. In the same way, there was a significant difference in ADF digestibility at (*p* < 0.05), among treatments.

**TABLE 4:** Dry matter and nutrient digestibility of Semien sheep fed hay and supplemented with cotton seed cake (CSC) and *Sesbania sesban* leaves.

Apparent digestibility (%)	Treatments						
	T1	T2	T3	T4	T5	SEM	SL
DM	59.69^c^	62.37^b^	63.86^ba^	64.42^a^	62.35^b^	0.43	⁎⁎⁎
OM	63.52^c^	65.55^b^	68.95^a^	69.06^a^	66.84^b^	0.51	⁎⁎⁎
CP	60.46^e^	69.41^d^	76.23^a^	74.78^b^	71.30^c^	1.27	⁎⁎⁎
NDF	50.55	52.07	51.9	53.16	50.36	0.38	ns
ADF	36.74^c^	39^ab^	38.32^ab^	41.38^a^	40.73^a^	0.55	⁎

*Note*: Mean values in a row having different superscripts (a–e) differ significantly; *** = significant at *p* < 0.001; * = significant at *p* < 0.05. T1 = only 100% CSC; T2 = 75% CSC + 25% *S. sesban*; T3 = 50% CSC + 50% *S. sesban*; T4 = 25% CSC + 75% *S. sesban*; and T5 = only 100% *S. sesban*.

Abbreviations: ADF = acid detergent fibre; CP = crude protein; DM = dry matter; NDF = neutral detergent fibre; ns = not significant; OM = organic matter; SEM = standard error of the mean; SL = significance level.

The apparent digestibility of the nutrient (DM, OM, CP, NDF and ADF) showed a slightly increasing trend as the level of inclusion of *S. sesban* (up to 50%) in the supplement increased at T2 and T3 (*p* < 0.001); when the inclusion amounts increased, nutrient (CP) digestibility decreased, which appeared at T4 and T5 (*p* < 0.001).

### 3.4 Body Weight Gain and Feed Conversion Efficiency

The IBW, FBW, BWC, ADG and FCE of Semien sheep are presented in Table 5. In the present study, IBW showed no statistically significant difference (*p* > 0.05) between treatments. The trend in live weight change of sheep over the experimental period shows a consistent increase throughout the experimental period.

**TABLE 5:** Body weight change and feed conversion efficiency of Semien sheep fed hay and supplemented with cotton seed cake (CSC) and *Sesbania sesban* leaves.

	Treatment feeds						
Parameters	T1	T2	T3	T4	T5	SEM	SL
IBW (kg)	15.37	13.97	15.67	15.50	15.30	0.21	ns
FBW (kg)	20.80^b^	20.50^b^	23.77^a^	22.57^a^	22.05^ab^	0.35	⁎⁎
TBWC (kg)	5.42^c^	6.52^b^	8.10^a^	7.07^b^	6.75^b^	0.22	⁎⁎⁎
ADG (g/day)	60.27^c^	72.49^b^	89.99^a^	78.60^b^	74.99^b^	2.52	⁎⁎⁎
FCE (g ADG/g/TDMI)	0.075^b^	0.082^b^	0.102^a^	0.082^b^	0.085^b^	0.002	⁎⁎

*Note*: a–c in a row having different superscripts differ significantly; ** = *p* < 0.01; *** = *p* < 0.001; T1 = only 100% CSC; T2 = 75% CSC + 25% *S. sesban* T3 = 50% CSC + 50% *S. sesban*; T4 = 25% CSC + 75% *S. sesban*; and T5 = only 100% *S. sesban*.

Abbreviations: ADG = average daily gain; FBW = final body weight; FCE = feed conversion efficiency; IBW = initial body weight; NS = not significant; SEM = standard error of the mean; SL = significance level; TBWC = total body weight change.

*S. sesban* at different levels of mixture with CSC has significantly (*p* < 0.01) increased the body weight gain of Semien sheep. The change in weight gain might be due to the potential of the *S. sesban* supplement for Semien sheep.

### 3.5 Partial Budget Analysis

The result of partial budget analysis for Semien lambs fed natural pasture hay as a basal diet and dried *S. sesban* leaf meal with CSC is shown in Table 6. The partial budget analysis was performed to evaluate the economic advantage of the use of *S. sesban* dried leave meal at different proportions instead of a commercial concentrate mixture of CSC.

**TABLE 6:** Partial budget analysis for Semien lambs fed as a basal diet *Sesbania sesban* dried leave meal with cotton seed cake.

	Treatment feeds				
Parameters	T1	T2	T3	T4	T5
Number of lambs	4	4	4	4	4
Purchase price of lambs (ETB/head)	2462.5	2337.5	2556.25	2587.50	2568
Purchase price of basal diet (ETB/kg)	9	9	9	9	9
Purchase price of CSC (ETB/kg)	34.5	34.5	34.5	34.5	
Purchase price of *S. sesban* (ETB/kg)	—	22	22	22	22
Total feed consumed(kg/head)	70.04	76.03	81.37	83.85	80.18
Total basal diet consumed (kg/head)	43.04	50.03	56.37	56.85	53.18
*S. sesban* dried leaf consumed (kg/head)	_—_	6.75	13.50	20.25	27
Cotton seed cake consumed (kg/head)	27	20.25	13.5	6.75	_—_
Cost of basal diet (ETB/head)	253	450	507	544	478
Cost of *S. sesban* dried leaf (ETB/head)	_—_	148.5	297	445.5	594
Cost of cotton seed cake (ETB/head)	931.5	698.63	465.75	232.88	_—_
Total cost of supplement (ETB/head)	931.5	847.13	762.75	678.38	594
Total feed cost (TVC) (ETB/head)	1184.5	1297.13	1269.75	1222.38	1072
Gross income (selling price of lamb ETB/head)	4550	4625	4850	4600	4500
Total return (ETB/head)	2087.5	2287.5	2293.75	2012.5	1932
Net return (ETB/head)	903	990.37	1024	790.12	860
ΔTVC	0	112.63	85.25	37.88	−112.5
ΔNI	0	87.37	121	−112.88	−43
MRR%	0	77	142	−297	38

Abbreviations: ETB = Ethiopian Birr; MRR = marginal rate of return; ΔNI = change in net income; ΔTVC = change in total variable cost.

## 4 Discussion

### 4.1 Chemical Composition of Experimental Feed

The CP content (6%) of the offered hay used in the present study had poor nutritional potential and might not support the maintenance requirements of sheep as it contains CP below the minimum level of 7%–7.5% required for microbial function (Gebremariam 2019). The high NDF content of the hay suggests, however, that the basal food intake may be restricted because NDF content was the primary factor limiting rumen fill and was directly associated with rumination or chewing time (Tesema et al. 2013). The low CP and comparably high NDF contents of the hay used in the present study could be attributed to the maturity of the mixed sward from which the hay was prepared. This result was in accordance with McDonald et al. (2002) who stated that advances in plant maturity were reported to be associated with low CP and high cell wall content. The CP content of hay used in the present study was in line with the study of Tesema et al. (2013) and Yirdaw et al. (2017b) who reported the CP (%) content of hay 6.23 and 6.04, respectively, but lower than the report of Bekele et al. (2013) which was 9.20%.

The NDF and ADF content of hay used in the present study was lower than the report of Kokeb et al. (2021) who stated that NDF was 61.12% and ADF was 45.65%. However, the present study was contradicted by the finding of Abebe and Tamir (2016) who stated that 66.66% and 55.55%, respectively.

The CP content of *S. sesban* leaf in the present study (25.13%) was within the range of 23.8%–31.7% indicated by Mekoya (2008) and comparable with the CP content of 25.3% reported by Michaele (2018). The CP content in the present study was also similar to Abreha et al. (2019), Bekele et al. (2013), Tekliye et al. (2018), who reported 26.8%, 26.37% and 26.4%, respectively, but the NDF (50.18%) and ADF (35.16%) values were higher than them (19.3%, 22.43%, 27.5%) and (15.5%, 19.64%, 20.2%), respectively.

The CP content of CSC used in the present study (27.28%) is slightly less than the report of Kahsu et al. (2021), Michaele (2018), Yirdaw et al. (2017b). CSC is excellent for fattening lambs and is nearly as effective as linseed meal (Michaele 2018). According to the report of Yirdaw et al. (2017a), the NDF content of 70% in a feed was thought to be enough to limit DM intake and digestibility, and the NDF values ranging from 35% to 42% were noted to have a relatively little impact on the intake and digestibility of DM. Thus, the level of NDF (49.78%) in CSC observed in the present study is expected to have some negative impact on the consumption and/or digestibility of the diets by the animals. Though the chemical analysis of the feed may not give full information regarding the availability of the nutrients present in the feedstuffs to animals, it was a good indicator of the quality of the feedstuffs (Yirdaw et al. 2017a). Feeds that contain 20% or more CP are classified as protein supplements (Kim et al. 2019). Therefore, results of the CP content of CSC and *S. sesban* were classified as high‐protein sources; however, hay was classified as poor‐quality feed.

### 4.2 DM and Nutrient Intake

The lower hay DM intake for T1 could be associated with the fact that CSC has relatively low rumen degradability due to its high fibre contents and is, therefore, a good source of bypass protein (Lata and Mondal 2021). On the basis of this idea and fact, as the animals get better bypass proteins, they will need lower roughage feed because microorganisms that are found in the rumen do not feed a large amount of protein, and the low intake in T1 might be due to the higher fibre fraction contained in the CSC.

In the present study, the total DM and OM intakes appeared to be highly impacted by the proportion of the supplemental DM intake. As such, total DM and OM intakes were the lowest for T1, intermediate for T2 and highest for the other two treatments, T3 and T4 (*p* < 0.01). The total DM and CP intake of T5 decreases to some extent. This is because *S. sesban* contains phenolic compounds (common anti‐nutritional factors tannins) that reduce voluntary feed intake (W. Bekele et al. 2013). The total NDF intake of experimental sheep was in the order of T4 > T5 = T3 = T2 > T1, and the ADF intake was in the order of T4 > T5 = T3 > T2 > T1, both of which appeared to be associated with increased levels of *S. sesban* supplementation. Even though there was no significant numerical difference, T1 had slightly lower CP intake as compared to the other four treatments. This slight variation in CP intake is associated with differences in the basal diet DM intake and fibrous content of the supplemental feed CSC. The total average CP intake of sheep in the present study was about 112.8 g/day; this value is higher than the 53.26–62.97 g/day reported by D. Tekle et al. (2016), Gizachew (2012), and slightly higher than the values of the CP intake that ranged from 54.23 to 111.98 g/day, reported by Worku et al. (2015). The differences in DM intake between treatments may be due to the variations in the type and amount of the basal diet as well as the supplement, growth stage of the animal and other similar factors (Animut and Tesema 2011). This slight variation in CP intake is associated with differences in the basal diet DM intake. The higher intake of NDF and ADF in all supplemented groups could mainly be attributed to the fibre content of the supplement. In general, the total DM intake of sheep in this study was about 830 g/day, which was comparable with the results of Tekliye et al. (2018), which was between 696 and 1015.72 g/day. However, the total DM intake noted in this study was higher than the report of Worku et al. (2015), 711 g/head/day, Kaffa sheep, and it was lower than 856–979 g/head/day reported by Yirga et al. (2022), for Hararghe Highland sheep.

### 4.3 Apparent Digestibility

Supplementation with different proportions of CSC and *S. sesban* significantly improved DM, OM, CP and ADF digestibility compared to T1, and this result is in agreement with the report of Wallie et al. (2012). It is possible that the supplements’ increased CP and decreased fibre content improved the ruminal microbes’ access to nutrients and microbial proliferation, with a resultant improvement in the total digestibility of dietary DM and nutrients. McDonald et al. (2010) reported that the addition of dietary protein to the supplement has increased protein availability for rumen microorganisms, speeding up the digestion process. According to Khalili and Varvikko (1992), dietary CP digestibility decreases with increasing *S. sesban* supplementation compared to concentrate supplementation because tannin reacts with protein to form a tannin protein complex. This reduces rumen fermentation and eventually depresses nutrient digestibility and voluntary feed intake (Frutos et al. 2004), and this happened to sheep intake on T4 and T5. In the current study, the CP digestibility of T5 (71.30%) was comparable with the findings of Tekliye et al. (2018) for higher supplemented (300 and 400 g *S. sesban*) groups and lower than the 85.8% reported by Worku et al. (2015) for Kaffa sheep and higher than the result (67.9%) of CP digestibility reported by Hagos (2014) for Tigray local sheep.

### 4.4 Body Weight and Feed Conversion Efficiency

Treatments with higher *S. sesban* percentages (T4 and T5) had higher DM intake than the other treatments but slightly lower average daily body weight gain. The reason is due to the deleterious effect of *S. sesban* on the growth of sheep when fed in large proportions (A. Mekoya 2008). There were no significant differences between T4 and T5 in BWC, ADG and FCE, which reflected that the supplements were comparable in their potential to supply nutrients to improve the weight gains of lambs. A similar trend has also been observed in BWC, ADG and FCE in T3 of the current study. The present study was comparable with the finding of W. Bekele et al. (2013), on the effect of substituting concentrate mix with *S. sesban* of Arsi‐Bale sheep, fed a basal diet of native grass hay. The ADG of lambs in T1 was comparable to the report of Yirdaw et al. (2017a), for Tigray highland sheep fed with a concentrate mixture of 62.8 g/day gain. The mean daily gain of lambs in T3 in the present study was also comparable with the report of Manaye (2005), ADG (83.3–99.8 g/day) for local sheep‐fed mixtures of 70%–90% Napier grass and 10%–30% supplementation with *S. sesban*. The average daily gain attained for T5 was higher than yearling Menz sheep (33.4–35.7 g/day) fed tef straw supplemented with sole *S. sesban* (Manaye et al. 2009) and lower than 103 g/day for sheep fed a diet containing 300 g/day *S. sesban* foliage (Moges et al. 2008) and 75.71 g/day for Abergelle rams supplemented with 300 g/day DM *S. sesban* (Tekle et al. 2016).

## 5 Conclusion

Generally, the total intake of DM and OM was higher in T4 > T3 = T5 > T2 > T1 and T4 = T3 = T5 > T2 > T1, respectively. However, the apparent digestibility of DM, CP, NDF and ADF for T3 was slightly higher than the other treatments. The results indicated that the DM, OM and CP intake and DM, OM and CP digestibility of the CSC *S. sesban* mixtures were higher than that of CSC alone. The CP intake increased as the level of *S. sesban* inclusion in the diet increased. The ADWG was higher in animals fed a diet containing an equal proportion of *S. sesban* with CSC as compared to the other treatments. The supplementation of *S. sesban* leaves to Semien sheep increased the total DM, CP and OM intakes. It also significantly improved the apparent digestibility of nutrients, average daily gain and feed conversion efficiency. Results of the partial budget analysis also demonstrated that sheep supplemented with CSC and *S. sesban* (T3) returned higher net income than T1, T2, T4 and T5. Therefore, from the results of the present study inclusion of *S. sesban* with CSC at equal proportions can respond to the biological performance and economic response of Semien sheep. However, further study should be done on why the effect of *S. sesban* feed beyond 50% did not respond to a better performance.

## Author Contributions

**Shewangzaw Addisu Mekuria**: supervision, conceptualization, methodology, writing – original draft, validation, investigation, formal analysis, writing – reviewing and editing, project administration. **Wondimu Demoz Tessema**: conceptualization, methodology, resources, investigation, software, data curation and writing – original draft preparation, formal analysis, reviewing, editing and funding acquisition. **Aschalew Assefa Teshager**: co‐supervision, conceptualization, methodology, validation, formal analysis, writing – reviewing and editing, supervision.

## Funding

The authors have nothing to report.

## Ethics Statement

For this experiment, ethical approval was obtained from the University of Gondar College of Veterinary Medicine and Animal Sciences Research Review and Ethics Committee (Ref. No. CVMAS 21/23).

## Conflicts of Interest

The authors declare no conflicts of interest.

## Data Availability

The data that support the findings of this study are available from the corresponding author upon reasonable request.
